# Protective effect of pomegranate flower extract against gentamicin-induced renal toxicity in male rats

**DOI:** 10.12861/jrip.2015.10

**Published:** 2015-06-01

**Authors:** Ferdos Sadeghi, Mehdi Nematbakhsh, Ali Noori-Diziche, Fatemeh Eshraghi-Jazi, Ardeshir Talebi, Hamid Nasri, Azam Mansouri, Aghdas Dehghani, Shadan Saberi, Soheila Shirdavani, Farzaneh Ashrafi

**Affiliations:** ^1^Water and Electrolytes Research Center, Isfahan University of Medical Sciences, Isfahan, Iran; ^2^Department of Biology, Falavarjan Branch, Islamic Azad University, Isfahan, Iran; ^3^Department of Physiology, Isfahan University of Medical Sciences, Isfahan, Iran; ^4^Isfahan MN Institute of Basic and Applied Sciences Research Center, Isfahan, Iran; ^5^Department of Clinical Pathology, Isfahan University of Medical Sciences, Isfahan, Iran; ^6^Department of Internal Medicine, Isfahan University of Medical Sciences, Isfahan, Iran

**Keywords:** Gentamicin, anate flower extract, Pomegr Nephrotoxicity

## Abstract

**Introduction:** Gentamicin (GM) as an antibiotic is used in clinic. However, its administration is limited by side effects such as nephrotoxicity. Herbal extracts could be used in therapeutic approaches.

**Objectives:** The present study was planned to investigate whether pomegranate flower extract (PFE) could ameliorate GM-induced renal toxicity in male rats.

**Materials and Methods:** Twenty eight male Wistar rats were divided into 5 groups. Groups 1 and 2 respectively received PFE 25 and 50 mg/kg for 9 days. Groups 3, 4 and 5 received saline, PFE 25 mg/kg, and PFE 50 mg/kg for 9 days, respectively, and GM (100 mg/kg/day) was administered from day 3 on. Blood samples were obtained, and after sacrificing the animals, the kidneys were removed for histopathology investigations.

**Results:** GM alone increased the serum levels of creatinine (Cr) and blood urea nitrogen (BUN), and tissue damage and kidney weight (*P* < 0.05). However, administration of low dose of PFE accompanied with GM decreased these markers significantly (*P* < 0.05). Low dose of PFE also ameliorated weight loss induced by GM (*P* < 0.05).

**Conclusion:** It is concluded that PFE 25 mg/kg is the effective dose to ameliorate nephrotoxicity induced by GM.

**Implication for health policy/practice/research/medical education:**

Pomegranate flower extract (PFE) may has protective role and ameliorate impact on nephrotoxicity induced by gentamicin.

## Introduction

Gentamicin (GM) is widely used in treatment of severe negative-gram infections (1-3). However, clinical administration of GM is accompanied with nephrotoxicity (4,5). GM leads to acute failure in kidney function (6), which is detected by lower glomerular filtration rate (GFR) and renal blood flow (RBF) (7), increasing serum creatinine (Cr) and blood urea nitrogen (BUN), and tubular necrosis (6). Generation of reactive oxygen species (ROS) (8) and membrane lipid peroxidation involve in nephrotoxicity (7). Studies have demonstrated that various compounds such as garlic (9), combination of vitamin E and selenium (10), and melatonin (11) decrease GM-induced renal injury. This is while herbal extracts could be used in therapeutic approaches (12-14). Pomegranate (*Punica granatum*) has antimicrobial, antioxidant (15), and anti-inflammatory (16) properties and can decrease the blood pressure (17). The advantageous properties of pomegranate on liver and kidney functions have been reported (15). Also, pomegranate extract could ameliorate the oxidative and histopathological damage induced by renal ischemia-reperfusion injury (18). This study was conducted to evaluate the effect of pomegranate flower extract (PFE) on GM-induced nephrotoxicity in rats.

## Materials and Methods

### Animals

Twenty-eight adult male Wistar rats (Animal Centre, Isfahan University of Medical Sciences) with the weight of 175.56±2.24 g were kept under standard conditions with free access to food and water. This research was approved in advance by the Isfahan University of Medical Sciences Ethics Committee.

### Preparation of pomegranate flower extract

Firstly, 500 g pomegranate flower was provided and powdered. Hydroalcoholic extract was prepared by ethanol: water (70:30) mixture using the percolation method. Hydroalcoholic extract was concentrated and dried to obtain 123 g pure powder.

### Measurement of total phenolic content of the extract

Total phenolic content of the extract was evaluated by the Folin-Ciocalteu method. Briefly, 20 µl extract 85% plus 1.58 ml deionized water and 100 µl Folin-Ciocalteu reagents were mixed. After 30 seconds, 30 µl Na_2_CO_3_ was added to the mixture. Then, this mixture was incubated at the temperature of 20^º^C for 2 hours. Finally, light absorbance was read at 765 nm. Total phenolic content of the extract was reported to be 9.98% gallic acid.

### Experiment design

The rats were divided randomly into 5 groups as follows: groups 1 and 2 (n = 6 for each) respectively received PFE 25 and PFE 50 mg/kg; intraperitoneally (i.p.) for 9 days. Group 3 (n = 5) received saline; i.p for 9 days and from day 3 on, GM 100 mg/kg was added. In groups of 1 and 2, saline was added to PFE from day 3 on. Group 4 (n = 4) received PFE 25 mg/kg; i.p for 9 days and from day 3, GM 100 mg/kg; i.p. was accompanied with PFE. Group 5 (n = 7) received treatment regimen the same as group 4, but the PFE dose was doubled. All animals were weighed daily. At the end of the study, the animals were anesthetized to obtain blood samples. After centrifuging the samples, the sera were removed and kept at -20^º^C until measurement. Finally, the animals were killed, kidneys were removed and weighed immediately. For pathological investigation, the left kidney was fixed in formalin. The right kidney was homogenized and centrifuged at 15000 g. The removed supernatant was kept at -20^º^C until measurement.

### Measurements

The serum levels of Cr and BUN were measured using diagnostic kits (Pars Azmoon, Tehran, Iran).The kidney level of nitrite (stable nitric oxide [NO] metabolite) was measured using ELISA kit (Promega Corporation, Madison, WI, USA). The kidney level of malondialdehyde (MDA) was measured by the manual method. Briefly, 0.5 ml of the sample was added to 1 ml trichloroacetic acid 10%. This mixture was centrifuged at 2000 g for 10 minutes. Then, 0.5 ml of the supernatant was mixed with 0.5 ml thiobarbituric acid 0.67% and placed in boiling water for 10 minutes. After cooling, the light absorbance was determined at 532 nm.

### Histopathological procedures

The left kidney was fixed in 10% neutral formalin and embedded in paraffin. After slicing, hematoxylin and eosin staining (H&E) was performed to examine the tubular atrophy, cast, debris, and necrotic materials in the tubular lumen. Intensity of tubular damage was scored from 1 to 4, while zero score was assigned to normal tissue without damage.

### Statistical analysis

Data were reported as mean ± SEM. The levels of BUN, Cr, MDA, and nitrite; and body and kidney weights were analyzed by one-way analysis of variance (ANOVA) followed by the Dunnett test. The groups were compared by the Kruskal-Wallis or Mann-Whitney U tests with regard the kidney tissue damage score (KTDS). *P*-values less than 0.05 were considered statistically significant. The software used for data analysis was SPSS version 16.

## Results

### Effect of PFE and GM on BUN and Cr serum levels and KTDS

**Figure 1:**
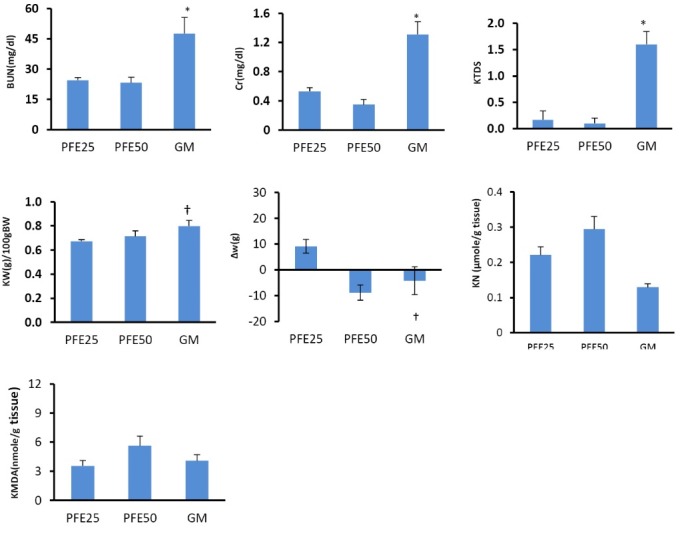
Blood urea nitrogen (BUN) and creatinine (Cr) serum levels, body weight difference (ΔW), kidney tissue damage score (KTDS), kidney weight (KW) g/100g bodyweight (BW), and kidney levels of nitrite (KN) and malondialdehyde (KMDA) in experiment groups. Groups of PFE 25 and PFE 50 received PFE 25 and 50 mg/kg for 9 days, respectively, and saline was added from day 3 on. The GM group received saline for 9 days and GM (100 mg/kg) was added from day 3 on.* indicates significant difference from the PFE 25 and 50 groups. † indicates significant difference from the PFE 25 groups (*P* < 0.05)

**Figure 2:**
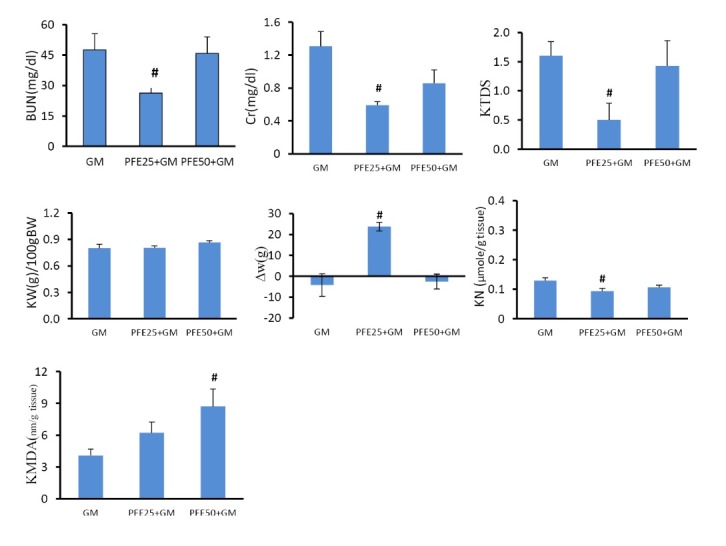
Blood urea nitrogen (BUN) and creatinine (Cr) serum levels, body weight difference (ΔW), kidney tissue damage score (KTDS), kidney weight (KW) g/100g bodyweight (BW), kidney levels of nitrite (KN) and malondialdehyde (KMDA) in experiment groups. Groups GM, GM+PFE 25, GM+PFE 50 respectively received saline, PFE 25 mg/kg, and PFE 50 mg/kg for 9 days, and GM 100 mg/kg was added from day 3 on. ^#^ indicates significant difference from the GM group (*P *< 0.05).

**Figure 3:**
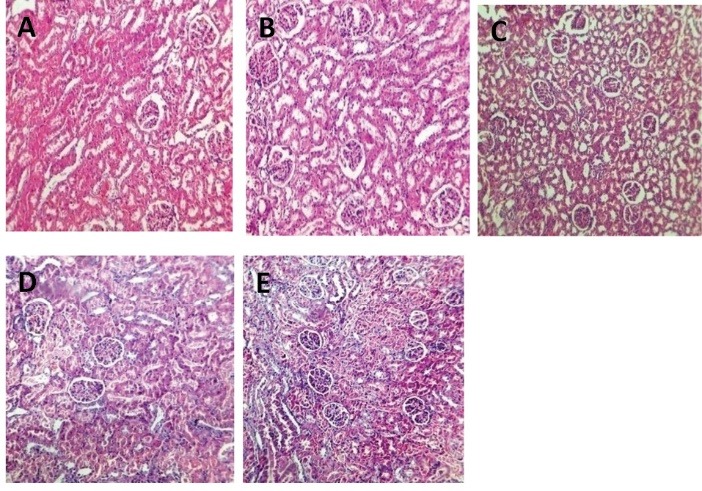
The pathology images (magnification: 100X) of kidney tissue in experimental groups. A-B received pomegranate flower extract (PFE) at the doses of 25 and 50 mg/kg for 9 days and saline was added from day 3 on. C-E received saline, PFE at the dose of 25 mg/kg and PFE at the dose of 50 mg/kg for 9 days, respectively, and GM 100 mg/kg was added from day 3 on.

GM alone significantly increased the serum levels of BUN and Cr when compared with the groups treated by PFE alone (*P *< 0.05). This is while administration of PFE 25 mg/kg plus GM decreased the serum levels of Cr significantly (*P *< 0.05) and BUN insignificantly (*P* = 0.1) when compared with the GM alone treated group. Administration of GM alone induced damage in renal tissue when compared with PFE treated alone groups significantly (*P *< 0.05). PFE 25 mg/kg reduced renal tissue damage induced by GM (*P *< 0.05) (Figures 1-3).

### Effect of PFE and GM on renal levels of MDA and nitrite

Administration of GM plus PFE (25 mg/kg) reduced the renal nitrite level significantly when compared with GM alone treated group (*P *< 0.05). Kidney level of MDA increased when GM plus PFE 50 mg/kg was administered (*P *< 0.05) (Figures 1 and 2).

### Effect of PFE and GM on body and kidney weights

GM itself increased kidney weight (KW) and induced bodyweight (BW) loss (*P *< 0.05). Administration of PFE 25 mg/kg ameliorated BW loss induced by GM (*P *< 0.05), but KW did not show significant difference in the groups treated with PFE 25 mg/kg plus GM, and PFE 50 mg/kg plus GM when compared with the GM alone treated group (Figures 1 and 2).

## Discussion

Nephrotoxicity is functionally characterized by rising in serum BUN and Cr levels (19) and decreasing GFR and RBF (7). Our results showed that GM induces renal tissue damage as well as increased serum levels of Cr and BUN. GM increases generation of reactive oxygen species (8), and induces loss of brush border resulting in enzymuria (20). In this study, the effect of administration of PFE at two different doses on nephrotoxicity induced by GM was investigated. The results obtained showed that low dose of PFE could decrease nephrotoxicity induced by GM, which was verified by reduction in BUN and Cr levels and KTDS. Similar to our results, other studies demonstrated that consumption of pomegranate extract decreases GM-induced nephrotoxicity (21). Protective properties of pomegranate have been documented in the literature (22). Aviram et al (23) reported that PFE consumption reduced serum levels of lipids and glucose and could attenuate atherosclerosis. Pomegranate phenolics including punicalagin, punicalin, gallic acid, and ellagic acid could reduce the antiatherogenic effects (23). It was shown in a study that different parts of pomegranate flower exhibit antioxidant activities in all free radical scavenging tests (24). Therefore, all parts of pomegranate flower could be considered as significant natural antioxidant sources (24). In the present study, it was determined that total phenol content of the extract is 9.98% gallic acid. It has demonstrated that administration of gallic acid has protective effects on renal markers, histopathology in vancomycin-induced nephrotoxicity in rats (25) and improves high fat diet-induced hyperlipidemia and fatty liver in mice (26).

NO, as an important agent in biologic processes (27), is produced by three NO synthase (NOS) isoforms; neuronal NOS (nNOS), endothelial NOS (eNOS), and inducible NOS (iNOS) (27,28). The present work showed that administration of PFE alone increases kidney nitrite level. One study indicated that pomegranate juice exhibits potent antioxidant activity and markedly protects NO against oxidative destruction via augmentation of the biological actions of NO. This is while the juice has effect neither on eNOS protein expression nor on the catalytic activity and does not elevate promoter activity in the eNOS gene (28). In another study, it has been demonstrated that both pomegranate fruit extract and pomegranate juice enhance the plasma level of nitrite and nitrate by increasing endothelial NO synthase (eNOS) expression (29). GM induces a decrease in kidney nitrite level. It is demonstrated that GM induces an increase and decrease in serum and urine levels of NO, respectively (27). Rivas-Cabañero et al (28) reported that administration of GM leads to expression of mac-iNOS mRNA in mesangial cells.

In our study, administration of PFE 50 mg/kg increased kidney MDA level in animals treated by GM. MDA is the final product of lipid peroxidation. Thus, this component has been known as an oxidative stress factor (30). Moreover, it is documented that antioxidant compounds ameliorate rising levels of renal MDA (31). However, we did not observe this. It is reported that administration of high doses of antioxidant components has toxic effects (32) and increases renal MDA level in renal damage induced by ischemia-reperfusion (33).

In the present study, it was observed that GM increased normalized kidney weight probably due to edema induced by acute tubular necrosis (34) and administration of PFE not only failed to decrease normalized kidney weight induced by GM, but also high dose of PFE intensified it nonsignificantly. Our results were in agreement with the findings reported by Erdem et al (34). Also, in the present work, it was observed that administration of PFE 50 mg/kg induces BW loss. Patel et al (35) showed that administration of 60 mg/kg/day pomegranate fruit extract induces a significant decrease in BW gain. It is reported that tannins reduce voluntary feed intake probably via inhibition of digestive enzymes (36). Therefore, pomegranate tannins in high dose may potentially diminish BW.

## Conclusion

It is concluded that PFE 25 mg/kg has protective role and ameliorate nephrotoxicity induced by GM.

## Authors’ contribution

FS conducted experimental procedures; MN planned and conducted the experimental procedures and data analysis, wrote and finalized it. FEJ, AM, AD, ShS, SS conducted experimental procedures. AT and HN conducted pathological diagnosis. AND and FA planned the experimental procedures and help in data analysis. All authors read and approved the final draft of the paper.

## Conflicts of interest

The authors declared no competing interests.

## Ethical considerations

Ethical issues (including plagiarism, misconduct, data fabrication, falsification, double publication or submission, redundancy) have been completely observed by the authors.

## Funding/Support

This research was funded by grant 292129 from Isfahan University of Medical Sciences.
